# Ultrasonic Vocalizations in Golden Hamsters (*Mesocricetus auratus*) Reveal Modest Sex Differences and Nonlinear Signals of Sexual Motivation

**DOI:** 10.1371/journal.pone.0116789

**Published:** 2015-02-25

**Authors:** Marcela Fernández-Vargas, Robert E. Johnston

**Affiliations:** Department of Psychology, Cornell University, Ithaca, NY, United States of America; Utrecht University, NETHERLANDS

## Abstract

Vocal signaling is one of many behaviors that animals perform during social interactions. Vocalizations produced by both sexes before mating can communicate sex, identity and condition of the caller. Adult golden hamsters produce ultrasonic vocalizations (USV) after intersexual contact. To determine whether these vocalizations are sexually dimorphic, we analyzed the vocal repertoire for sex differences in: 1) calling rates, 2) composition (structural complexity, call types and nonlinear phenomena) and 3) acoustic structure. In addition, we examined it for individual variation in the calls. The vocal repertoire was mainly composed of 1-note simple calls and at least half of them presented some degree of deterministic chaos. The prevalence of this nonlinear phenomenon was confirmed by low values of harmonic-to-noise ratio for most calls. We found modest sexual differences between repertoires. Males were more likely than females to produce tonal and less chaotic calls, as well as call types with frequency jumps. Multivariate analysis of the acoustic features of 1-note simple calls revealed significant sex differences in the second axis represented mostly by entropy and bandwidth parameters. Male calls showed lower entropy and inter-quartile bandwidth than female calls. Because the variation of acoustic structure within individuals was higher than among individuals, USV could not be reliably assigned to the correct individual. Interestingly, however, this high variability, augmented by the prevalence of chaos and frequency jumps, could be the result of increased vocal effort. Hamsters motivated to produce high calling rates also produced longer calls of broader bandwidth. Thus, the sex differences found could be the result of different sex preferences but also of a sex difference in calling motivation or condition. We suggest that variable and complex USV may have been selected to increase responsiveness of a potential mate by communicating sexual arousal and preventing habituation to the caller.

## Introduction

Animal vocalizations have the potential to encode functionally relevant information for a receiver. This information is usually encoded by some kind of variation in spectral and temporal characteristics in the vocal signal or by differences in the time or frequency of use of specific sounds. Vocal signatures and call recognition have been demonstrated in audible vocalizations produced by several species of mammals (e.g. [1–3]). In addition, many mammal species have evolved to produce sounds over a wide range of frequencies in different environments. The production of ultrasonic vocalizations (USV) in rodents is an example of this, and currently, at least 50 species in 30 genera of rodents are known to produce USV [4]. Adult rodent USV have been typically recorded during intersexual encounters in several species, are activated by gonadal steroids and appear to facilitate mating, supporting the interpretation that these signals may function in courtship and mating behaviors [5–31]. In addition, some vocal signatures have been found in house mice USV that serve to discriminate vocalizations from mice of different ages [32,33], natural populations [34], strains [35–37] and individuals [26,31,38].

It is unclear, however, if USV could be used to discriminate sex in rodents. In non-rodent vertebrates, it is common that one sex, but not the other, produces vocalizations or that each sex produces distinctive calls (e.g. [1, 39]). These are known as sex-specific vocalizations [40]. In other species, both sexes produce the same type of call but they differ in calling rates, in the context in which the call is used or in the acoustic structure of the calls. These are known as sex-typical vocalizations [40]. Thus, male and female vocal behavior can differ in the diversity of repertoire and usage. The production of USV has been reported for female as well as male rodents but there has been variation in the context used for females (e.g. resident/intruder paradigm vs. neutral arenas, same-sex vs. opposite-sex interactions) [34,41–46]. Also, males have been found to be the predominate callers during intersexual contact in some species [5,6,14,15,41]. Few experimental studies have compared both sexes’ vocal behavior during the same sexual context and when the identity of the caller is unambiguous [34,45]. No discriminatory signatures in USV were found between sexes of free-living California mice [45]. Because this species is monogamous, however, sex differences in the vocal behavior were not necessarily expected [45].

In addition, mammalian vocalizations are often considered an index of behavioral state that can be actively modulated by motivation and arousal [47,48] and can also significantly influence the affective state and behavior of others [49]. Currently, specific acoustic properties of USV have been proposed to convey varying degrees of motivational states. For instance, USV produced by rats are considered a model of the relationship between vocalizations and affective states of the callers. Adult 22kHz are associated with aversive events whereas adult frequency modulated (FM) 50kHz calls are associated with positive social interactions [48,50,51]. The avoidance of 22kHz and the approach behavior to 50kHz USV are believed to reflect appetitive affective states and different rewarding values of the signals [50,52–54]; contrary results found in [55,56]. Moreover, the presence of nonlinear phenomena (NLP) in mammalian sounds and their functional significance in communication has received increased attention and study in recent years [57–62]. When the oscillations of the sound source (e.g. vocal cords) are not periodic and out of synchrony, irregular perturbations to the spectral structure, or NLP, are observed in vocalizations [58]. These phenomena were previously overlooked and once thought to be present in just a few irregular variants that were difficult to analyze and quantify [57]. Today, NLP are known to be quite common in many vertebrate vocalizations, somewhat related to affective state and physical condition and responsible for increasing the complexity of the vocal repertoire [63–67].

Although USV in laboratory rats and mice have been systematically studied, especially in the last decade, and there is a better understanding of this vocal behavior [29,31,34,46,53,68–70], the evolution and function of USV in rodents remains unclear. A role in social communication is described as the general function, but there are species differences. Thus, variability in function is expected depending upon the social structure, mating system and evolutionary history of each species. Consequently, comparative analyses will provide a more useful insight about the biological significance of this behavior that is so widespread in this taxonomic group. In addition, a better understanding of this natural behavior will provide more opportunities to explore the neural mechanisms behind the production and perception of social signals.

In the golden hamster (*Mesocricetus auratus*), both males and females show high rates of USV during and after interacting with opposite sex conspecifics or just their odors [7,20]. The function of these vocalizations is not known but it could be hypothesized that they facilitate sex recognition and/or mate assessment. Under semi-natural conditions, golden hamsters lived solitarily in simple burrow systems, and social contact was usually restricted to mating interactions, when the female briefly allowed the male access to her burrow [71,72]. Males competed for access to receptive females and sperm competition has been demonstrated after multiple matings [72,73]. Also, females are aggressive and dominant toward males during the non-estrous days of the cycle [71,74]. Therefore, sex-specific or sex-typical variation in vocal signals could facilitate sex recognition, allowing males to identify receptive females and perhaps allowing females to select males around the time of mating. Therefore, in the present study, we examined in detail the repertoire of USV produced after a social interaction and tested whether vocal behavior was sexually dimorphic. Specifically, we examined sex differences in: 1) calling rates, 2) composition, by quantifying the occurrence of calls of varying structural complexity, distinct call types and nonlinear phenomena (NLP) and 3) spectro-temporal characteristics of the USV most commonly used. In addition, we analyzed the variability in acoustic parameters of the USV of individual hamsters to determine whether that variability carries information about the identity of the caller. It is important to emphasize that in this study we analyzed and categorized the vocal repertoire of females of a known estrous state, produced in the same sexual context as in males and when the identity of the caller was unequivocal.

## Material and Methods

### Subjects and animal housing

All animal procedures in this study were conducted with approval from Cornell University’s Institutional Animal Care and Use Committee (protocol #1993–0120). Animals were bred and raised in captivity at Cornell University. Hamsters were weaned at 30 days of age and housed individually in solid bottom polycarbonate cages (45 x 24 x 14.5 cm) with sani-chip bedding material inside a colony room. Food and water were available *ad libitum*. Hamsters were maintained on a 14L:10D light:dark schedule. Behavioral experiments were run between 13:00 and 16:00h (middle of dark phase) inside a sound proof room under a low dim light.

We divided the hamsters used in this study into two groups: subject and stimulus animals. We used 28 adult male and 26 female hamsters (6–10 months old) as subjects that were sexually mature but had never had social experience after weaning or breeding experience. We randomly selected pairs of subject-stimulus hamsters, controlling relatedness and familiarity. Each subject and stimulus animal was used only once in behavioral trials. The estrous cycle of all the females in the colony was regularly determined after sexual maturity by briefly placing a stud male in the female’s home cage and watching for lordosis. Female hamsters have a very consistent 4-day estrous cycle [75], so the estrous day of each female used in the study was verified using the stud male four days before the testing day. During the experiment, a female’s estrus was again confirmed by observing the female displaying lordosis during the interaction with a male across the wire mesh.

### Experimental procedure: Social elicitation of USV

We used a Plexiglas box (91.5 x 50.5 x 30.5 cm) lined inside with acoustic foam (SONEXone acoustical panels, 5.08 cm thick). Inside this box, we had a rectangular enclosure made of wire mesh (41 x 21 x 29.5 cm) with a wire mesh barrier in the middle dividing the enclosure in two compartments.

Our behavioral experiment consisted of male and estrous female subjects interacting with a same or opposite sex stimulus. Each experimental trial consisted of 3 periods, each lasting 3 min. Using a plastic cup, we transferred the subject into one of the compartments of the wire mesh enclosure. After a 2 min acclimation period, the *pre-interaction period* started and the subject moved freely inside the compartment. At the end of the first 3 min, we introduced the stimulus hamster inside the other compartment and the hamsters interacted across the mesh divider for 3 min. The barrier allowed visual, olfactory and acoustic interactions, while restricting physical contact. At the end of this *interaction period*, we removed the stimulus animal and the subject remained in its compartment for an additional 3 min (*post-interaction period*). We performed four different permutations of this experiment using different combinations of subject and stimulus hamsters. Between trials, all equipment was cleaned and rinsed with 50% ethanol to eliminate residual odors.

During each period, we recorded all the USV produced using SONOTRACK 1.5.0 (Metris B.V., Netherlands), at 200 kHz sampling rate and 16-bit sample resolution. We used three microphones (SONOTRACK, 15–100 kHz) to record simultaneously from different locations during each period. We placed microphones 1 and 2 in front of the subject’s compartment at a height of 10 cm. Microphones 1 and 2 were 23 and 55 cm, respectively, away from the subject’s compartment. Microphone 3 was placed 45 cm above the subject’s compartment.

To determine whether the vocal repertoires of male and female hamsters were different, we analyzed the USV recorded in the post-interaction period of opposite-sex experiments only. These vocalizations were elicited by both sexes after the same social experience and under the same context. The USV produced during the social interactions were not used to examine sex differences because estrous females displayed lordosis in the presence of a male and female hamsters do not produce vocalizations during lordosis [7,21]. During the short periods when the female was not in lordosis, we could not analyze the USV produced due to uncertainty of the identity of the caller. SONOTRACK 1.5.0 does not provide real-time spectrogram display, thus at the moment of collecting the data, we were not confident enough to determine when each member of the pair was vocalizing. Given that during the pre-interaction period the numbers of USV produced were very low, the vocalizations from this period were not analyzed either. Therefore, with the exception of calling rates, all the analysis of USV was performed on those vocalizations recorded during the post-interaction period, when the identity of the caller was unequivocal.

### Data analysis

The acoustic structure of USV had been previously studied in golden hamsters [76], but the complete repertoire and its variability demanded a more thorough acoustic analysis and description. Given that the general patterns of these vocalizations were not known, we avoided using automatic computer counters and performed the categorization manually by visual inspection of the spectrograms. All USV recorded by the SONOTRACK recorder were converted into .wav files and analyzed using Raven Pro 1.4 (Cornell Lab of Ornithology, Ithaca, NY, U.S.A). We generated spectrograms in Raven with a Hanning (Hann) window function, 16-bit sample format, a DFT (discrete Fourier transform) size of 512 samples and a time window overlap of 80%. In addition to the acoustic measurements calculated by the software, we annotated each spectrogram with call ID number, call type, structural complexity and nonlinear phenomena (NLP), each defined below.

We divided our analysis of sex differences in vocal repertoire into three main areas: calling rates, composition and acoustic structure. For the analysis of calling rates, we included all male and female subjects used in the opposite-sex experiments. However for the analysis of composition and acoustic structure, we excluded 4 males and 5 females because they emitted fewer than 18 calls in the post-interaction period. Eighteen calls was the lower quartile of the distribution of total calls emitted by subjects after interacting with an opposite sex hamster. A sample size of less than 18 calls per individual was considered not a representative sample of calls for statistical analysis and less likely to provide enough high quality calls for spectrogram analysis.

**Calling rates**. We counted the total number of USV uttered during the 3 min recording period by visually inspecting the spectrograms. We excluded short sounds that had a duration less than 10 ms. The USV used for acoustic analysis were those recorded in the microphone with the highest filtered root-mean square (F-RMS) amplitude, which had to be at least double the F-RMS amplitude of the background noise (i.e. good signal-to-noise ratio). USV overlapped by object sounds (e.g. noise from the mesh cage) were counted in the total number of calls produced but were not included in the sound analysis. A call surrounded by more than 150 ms of silence was identified and counted as an individual USV (see below).

**Composition**. General composition of the repertoire was described based on structural complexity, call types and the presence of NLP. The proportion of calls produced under the different categories of complexity, call types or NLP was calculated over the total recording time to provide a percentage of occurrences of these different components.

*Complexity* described the structural complexity based on the number of notes present in an individual call. We defined a note as a single, continuous and complete spectrographic contour. A 1-note simple call consisted of one note and its harmonically related elements and resonances. A composite call was counted as a single unit or call that consisted of 2 or more notes separated by less than 150 ms and more than 10 ms of silence. The silence between two notes in a composite call is roughly similar to the average duration of any 1-note simple call (∼150 ms). This criterion was previously used in the categorization of bat calls [77]. If two or more notes were separated by less than 10 ms of silence, those were classified as 2-note, 3-note or 4+note simple calls. These calls, composed of notes separated by such a small amount of time, were essentially frequency jumps that we will describe later.

**Fig 1:**
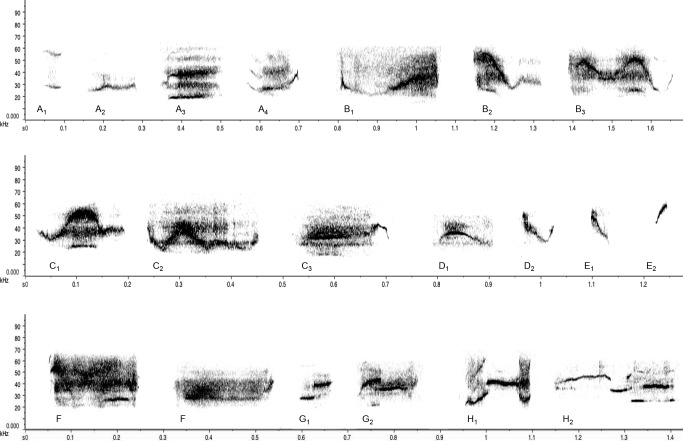
Spectrograms of typical call types produced by golden hamsters after interacting with a stimulus hamster of the opposite-sex. Call subtypes are merged into 8 major categories of call types: **A**. Flats (Flat^1–3^, 2tail-flat^4^), **B**. Waves (Wave down-up^1^, Wave up-down^2^, Complex wave^3^), **C**. Humps (Center hump^1^, Left hump^2^, Right hump^3^), **D**. Chevrons (Chevron-down^1^, Chevron-up^2^), **E**. Sweeps (Down-sweep^1^, Up-sweep^2^), **F**. Broadband, **G**. 1-Frequency jump (1-Jump up^1^, 1-Jump down^2^), **H**. Multi-Frequency jumps (2-Step^1^, 3+Step^2^).

**Call type categorization**. We classified hamster USV into morphologically distinct types based on the shape of the frequency trajectory or contour. We adapted the terminology used for this categorization from the descriptions of vocalizations made for other mammal species, especially those used for USV in the house mouse and in the rat [33,36,68,78–80]. Rodent USV have been described as flat or constant frequency (33,36,68,78–80), wave frequency modulation (35,36,80), hump frequency modulation (79), chevrons (or U-shaped or inverted-U modulation; 33,35,36,68,79,80), frequency-modulated sweeps (or upward and downward; 35,36,68,79,80) and frequency steps or jumps (1, 2 or multi-steps, 33,35,36,68,79,80). We identified these types in the USV produced by hamsters and classified them further into a more detailed scheme of 21 categories of call subtypes to categorize as much of the variation as possible (Table 1, Fig. 1).

**Table 1:** Description of the all the call types used to categorize USV in male and estrous female golden hamsters (*Mesocricetus auratus*).

Complexity	Call Types	Subtypes	Description
Simple 1-note	Flats	Flat (FT)	Near- constant frequency with < 5 kHz of modulation
		Erratic flat (EF)	Flat signal with random inflections < 5 kHz along the main frequency
		Tail-flat (tailFT)	Flat signal with an upward or downward inflection at the beginning.
		Flat-tail (FTtail)	Flat signal with an upward or downward inflection at the end.
		2tail-flat (2tailFT)	Flat signal with an upward or downward inflection at the beginning and at the end.
	Waves	Wave up-down (WUD)	Frequency increases > 5 KHz from starting point to the first frequency peak and then decreases > 5 KHz into one frequency bottom.
		Wave down-up (WDU)	Frequency decreases > 5 KHz from starting point to the first frequency bottom and then increases > 5 KHz into one frequency peak
		Complex wave (CW)	Wave containing 2 or more frequency peaks or bottoms.
	Humps	Left hump (LH)	Frequency is constant and then increases > 5 KHz from into a frequency peak or hump that decreases into constant frequency. Hump forms at the left side, closer to the beginning of the call.
		Right hump (RH)	Frequency is constant and then increases > 5 KHz from into a frequency peak or hump that decreases into constant frequency. Hump forms at the right side, closer to the end of the call.
		Center hump (CH)	Frequency peak or hump is approximately at the center of the call. Sides are constant frequency
	Chevrons	Chevron-up (CU)	Upright chevron-shaped (U-shape) note
		Chevron-down (CD)	Downright chevron-shaped (inverted U-shape) note
	Sweeps	Down-sweep (DSW)	Frequency decreases > 5 KHz from starting point to end
		Up-sweep (UPS)	Frequency increases > 5 KHz from starting point to end
	Broadband	Broadband (BB)	Noisy, atonal call with unclear frequency bands and the contour cannot be indentified as any particular type
Simple 2-notes	1-Frequency jump (1-FJ)	1-Jump up (1JU)	Sudden frequency change to a higher frequency. Elements separated by <10 ms of silence
		1-Jump down (1JD)	Sudden frequency change to a lower frequency. Elements separated by <10 ms of silence
Simple multi-notes	Multi—Frequency jumps (multi-FJ)	2-Step (2S)	Two sudden frequency changes to a higher and then a lower frequency. Elements separated by <10 ms of silence
		Reversed 2-Step (R2S)	Two sudden frequency changes to a lower and then a higher frequency. Elements separated by <10 ms of silence
		3+Step (3+S)	Three or more sudden frequency changes. Elements separated by <10 ms of silence

The *Nonlinear phenomena (NLP)* classification quantified the occurrence of nonlinearities in the repertoire. Although several NLP have been described in the literature, we mainly found two types [57,81]. First, vocalizations were produced as a broadband spectrum with energy at many frequencies and traces of harmonic elements. This is usually termed as deterministic chaos (DC) [57,82]. Given that the presence of DC was widespread, we identified by visual examination four levels of irregularity that fell on a scale of increasing atonality. The category Linear (L) referred to tonal USV, with a clearly defined frequency contour and without major traces of noise. Deterministic chaos 1 (DC1) described harmonic calls with sudden onset/offset of chaotic noise and remnants of harmonic structure. Deterministic chaos 2 (DC2) referred to harmonic calls with significant noisy overlay and energy concentrated in one or few bands along the whole call. Deterministic chaos 3 (DC3) consisted of noise without clearly distinguishable frequency bands and with energy distributed approximately in all frequencies. This classification did not quantify the degree of irregularity or determine whether atonal calls were low- or high-dimensional [82]. However, this classification provided a general overview of the presence of chaos in male and female USV. The second type of NLP that we found in hamster USV was a sudden frequency change in the peak carrier, disrupting and moving the pitch up or down. These abrupt changes are termed frequency jumps (FJ) and are believed to be the result of instability in the vibratory pattern [81].

**Acoustic Structure**. For the spectrogram analysis, we selected a total of 1103 1-note simple calls that had good signal-to-noise ratio (at least double the F-RMS of the background noise) including the principal carrier, harmonics and resonances. Here, we defined the principal carrier as the main frequency contour of the highest amplitude. We avoided using the term fundamental frequency (F0) because in many cases the frequency with the highest amplitude was a harmonic and not the F0. From those 1103 simple calls, only in 540 calls was the carrier identifiable so that it was possible to extract the principal carrier. Therefore in the rest of this paper, when we make reference to the principal carrier, this is the F0 or the harmonic of highest (or peak) amplitude.

We measured 14 spectro-temporal parameters in Raven. The descriptive statistics (mean ± standard error) shown in Table 2 were calculated on the means per individual. In addition to the commonly used acoustic parameters, we included two measures of entropy, which measured the energy distribution within a selection (a high value means greater disorder in the sound while a zero value means a sound with energy in only one frequency bin). Average entropy calculated the entropy value for each slice of time and then took the average, while the aggregated entropy measured the total energy within a selection providing the overall disorder in the sound [83]. Inter-quartile range bandwidth (IQR BW) contained the middle 50% of the energy in a selection. We analyzed the principal carrier of all 1-note simple call types combined (*N* = 540 calls) and then subdivided by flats (*N* = 319), waves (*N* = 103), humps (*N* = 57) and sweeps (*N* = 31). Additionally, we analyzed the spectro-temporal parameters of 2-note simple (*N* = 149) and multi-note simple *(N* = 219) call types.

**Table 2:** Acoustic parameters (X̅ ± SE) measured for the analysis of the entire full spectrum of simple 1-note calls and of the principal carrier of simple 1-note calls produced by male (*N* = 14) and female (*N* = 12) subjects after interacting with a stimulus hamster of the opposite sex.

	Analysis full spectrum (*N* = 1103)			Analysis of the principal carrier (*N* = 540)		
	Male	Female	*P*-value^f^	Male	Female	*P*-value^f^
Duration (ms)	106.5 ± 4.96	130.1 9.3	< 0.05	99.3 ± 5.31	123.6 ± 11.9	NS
Minimum frequency (kHz)	23.0 ± 0.71	20.1 ± 0.42	< 0.01	26.4 ± 0.88	23.5 ± 0.68	< 0.05
Maximum frequency (kHz)	54.5 ± 0.97	54.3 ± 1.00	NS	40.3 ± 1.07	40.9 ± 0.87	NS
Q1 frequency (kHz) ^a^	33.5 ± 0.99	32.3 ± 0.73	NS	33.1 ± 1.09	31.1 ± 0.75	NS
Q3 frequency (kHz) ^b^	40.6 ± 0.89	39.5 ± 0.9	NS	35.7 ± 1.05	34.8 ± 0.84	NS
Center frequency (kHz)	36.7 ± 0.94	35.6 ± 0.85	NS	34.5 ± 1.08	32.9 ± 0.77	NS
Peak frequency (kHz)	36.4 ± 1.01	35.4 ± 0.85	NS	34.7 ± 1.05	33.2 ± 0.81	NS
Delta frequency (kHz) ^c^	31.5 ± 1.23	34.2 ± 1.17	NS	13.9 ± 0.65	17.4 ± 0.49	<0.001
IQR BW (kHz) ^d^	7.15 ± 0.78	7.20 ± 0.33	NS	2.62 ± 0.23	3.71 ± 0.24	<0.01
Average power (dB)	66.4 ± 0.80	65.5 ± 0.83	NS	68.3 ± 0.90	66. 6 ± 0.90	NS
Peak power (dB)	84.1 ± 0.84	83.7 ± 0.84	NS	83.7 ± 1.05	82.7 ± 0.94	NS
Energy (dB)	100.6 ± 0.72	100.9 ± 0.86	NS	98.6 ± 0.95	98.5 ± 1.10	NS
F-RMS Amplitude (u) ^e^	992.9 ± 82.1	939.6 ± 75.3	NS	861.2 ± 105.7	771.9 ± 77.5	NS
Aggregated Entropy (u)	5.06 ± 0.12	5.24 ± 0.08	NS	4.11 ± 0.09	4.41 ± 0.08	< 0.05
Average Entropy (u)	4.52 ± 0.10	4.60 ± 0.10	NS	3.53 ± 0.07	3.76 ± 0.09	< 0.05

^a^ 1st quartile frequency

^b^ 3rd quartile frequency

^c^ difference between the upper and lower frequency limits of the selection

^d^ inter-quartile range bandwidth

^e^ filtered root-mean-square amplitude.

^f^ Statistical comparisons made by two-group Kruskal-Wallis test

To measure the degree periodicity in the hamster USV, we quantified the harmonic-to-noise ratio (HNR). HNR is the ratio of periodic components to noise, finding the maximum difference in dB between peak of any present harmonic and the averaged chaotic energy in the selection of the spectrogram for each call. Lower HNR values indicate increased aperiodicity and calls are perceived as harsh and hoarse, while higher HNR values indicate high tonality and calls are perceived as harmonic and clear [84,85]. This measure does not identify turbulent noise or differentiate between different degrees of deterministic chaos but it has been demonstrated to be useful for quantifying the noise within a signal [85]. Moreover, HNR was positively correlated with a low-dimensional nonlinearity measure used to distinguish different degrees of atonal calls [82]. To measure this parameter, we randomly selected 16 1-note simple calls from the post-interaction recordings of each subject and extracted their HNR values using the “To Harmonicity (cc)” command (0.01 s time step, minimum frequency 10000 Hz) in the sound program PRAAT 5.3.55 [86].

We assessed patterns of call variability within- and among-individuals by calculating the coefficient of variation (CV = (SD/mean)*100) for each acoustic parameter for the data set from 12 males and 14 females. We estimated within-individual variation (CV_w_) for each individual and the among-individual variation (CV_a_). The CV_w_ was calculated based on the individual means and SD. The CV_a_ was calculated from the grand mean and SD of each parameter based upon the average values per individual (averaging 26 individual means). We then estimated the ratio of CV_a_ to CV_w_ (CV_a_/CV_w_) as a measure of relative inter-individual variability. This ratio is often referred to in some studies as the potential for individual coding (PIC) (e.g. [87,88]). A ratio or a PIC > 1 would indicate that the variability between individuals is greater than the variability within individuals, and therefore, that the parameter could function as an individual signature.

### Statistical analysis

All tests were two-tailed and used a threshold significance level of P < 0.05. We tested for normality using the Shapiro-Wilk Normality test in JMP 9 (SAS Institute). We performed nonparametric tests in those cases in which the data failed to meet the assumptions of normality and heteroskedasticity and when data transformations were not successful in normalizing the data. We compared the number of USV between the pre- and post-interaction using a Wilcoxon signed-rank test for each combination of subject and stimulus hamsters. We used a Kruskal-Wallis test to compare the post–interaction calling rates between the same and opposite sex stimulus hamsters in each subject sex. We explored differences in calling rates along the 3 min of the post-interaction recording period using a Friedman test.

To statistically analyze the data on composition, we obtained the proportion of calls produced per individual (number of calls in each category for each subject / total number of calls recorded in each subject) and averaged them by category within complexity, call type or NLP. We performed an angular (arcsine) transformation of the proportions and used those as the response variable in a generalized linear model (GLM) testing the main effects of composition (either complexity, call types or NLP), sex and the interaction between the two. Main effect pairwise comparisons of parameter estimates were made by JMP using a Student’s *t* test and a Bonferroni correction for multiple comparisons. We measured repertoire diversity by calculating a Shannon-Wiener index [89] for each subject based on the 21-call subtype categorization (Table 1) and then comparing them between males and females with a Student’s *t* test. The Shannon Diversity Index computes the number of different call subtypes in each individual and the evenness of the distribution.

We performed a principal component analysis (PCA) to reduce the number of parameters into a few orthogonal variables (principal components, PC) that would explain most of the variation in the data and ran a generalized linear mixed model (GLMM) with each PC as the dependent variable. The GLMM included subject ID as a random effect to account for repeated sampling of the same individuals (several USV per individual) and sex as a fixed effect. In the cases in which the normality assumption was not fulfilled, we took mean values of the vocalizations from the same individual and performed a Kruskal-Wallis test for two groups with a chi-square test statistic to compare parameters between males and females. Similarly with those mean values, we used Spearman correlations to explore whether call rates or call duration predicted the variation in other acoustic parameters. We also tested the effect of sex in HNR values (log-transformed to reach normality) using a GLMM. Kernel plots of male and female HNR distributions were constructed in “R” statistical package version 2.2–5.3 (R development Core Team 2013) on the means per individual (12 females, 13 males) instead of total calls from males (*N* = 189) and females (*N* = 191) to avoid pseudoreplication. Skewness, kurtosis and the coefficient of variation (CV) were calculated on male and female distributions and compared with a Student’s *t* test. Finally, we explored whether changes in log HNR were predicted by other acoustic parameters using a GLMM.

In addition to calculating the ratio of CV_a_ to CV_w_ to examine individual variability, we performed a stepwise forward discriminant function analysis (DFA) in SPSS v.*19*.*0* (SPSS Inc, Chicago, IL) to determine whether each call could be correctly assigned to the individual caller. The prior probabilities were computed from group sizes. We examined correct classification using a leave-one-out cross-validation procedure in which each call is assigned to each individual based on the discriminant functions established with all calls except with the one being classified, and thus, testing for predictive power and the probability of misclassification.

## Results

### Calling rates

**Fig 2:**
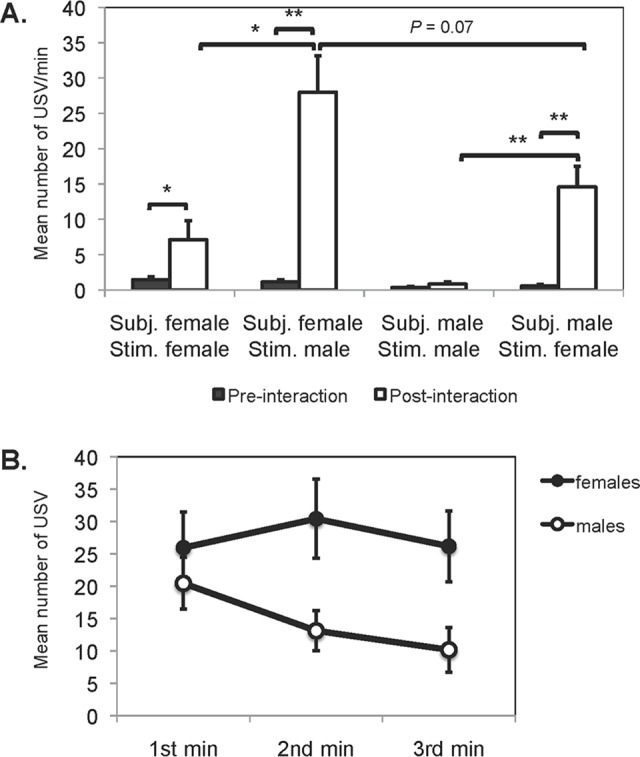
Calling rates. (A) Mean (± SE) number of USV emitted by male and estrous female golden hamsters before and after an interaction with a same or opposite sex stimulus hamster. (B) Mean (± SE) number of USV emitted across the recording period after an interaction with a stimulus hamster of the opposite sex. *P ≤ 0.05, **P ≤ 0.01.

The series of social elicitation of USV experiments showed that subjects rarely produced USV in the *pre-interaction period*, but produced a higher number of USV in the *post-interaction period* after male subjects (Wilcoxon signed-rank test: *W* = 68, *P* < 0.001, *N* = 18) and female subjects (*W* = 75.5, *P* < 0.001, *N* = 17) interacted with an opposite sex stimulus animal (Fig. 2A). There was also a significant difference between the total number of USV produced between pre- and post- interaction periods in female subjects (*W* = 15, *P* = 0.04, *N* = 9), but not in male subjects when interacting with a same sex stimulus individual (*W* = 13.5, *P* = 0.117, *N* = 10) (Fig. 2A). By comparing the post-interaction calling rates, we found that encounters with the opposite sex, and not with the same sex, elicited the largest amount of USV in male (Kruskal-Wallis test: *X* ^*2*^ = 12.6, *P* < 0.01) and in female subjects (Kruskal-Wallis test: *X* ^*2*^ = 6.02, *P* = 0.0141) (Fig. 2A). Thus, an interaction and removal of a conspecific elicited the production of USV in hamsters, except in male-male interactions. The interaction between opposite sexes was the strongest social interaction for eliciting vocal behavior (Fig. 2A).

We examined a potential sex difference in calling rates after the interaction with an opposite sex hamster and found that female subjects did not call significantly more than male subjects (Kruskal-Wallis test: *X* ^*2*^ = 3.17, *P* = 0.0746) (Fig. 2A). There was high individual variability, however. For instance, the total number of calls per individual in a 3-min recording ranged between 2–208 in females (CV 75.5%) and 0–141 in males (CV 86.3%). Most individuals ranged between 18–96 vocalizations (IQR of both sexes pooled together). Five (29%) females and 4 (22%) males called fewer than 18 times and could be considered as “low callers”. On the other hand, 7 (41%) females and 2 (11%) males called more than 96 times and those individuals could be considered as “high callers”. Thus, it seemed that females were more likely to be high callers than males, which might explain the sex difference trends in Fig. 2A. But on average both sexes were capable of producing similar numbers of calls within this post-interaction context. In addition, this range provided a useful criterion for the behavioral screening of subjects for future experiments examining possible factors modulating the frequency of this behavior.

Finally along the 3 min of the post-interaction period, we found that during the first minute, both male and female subjects vocalized at relatively high rates after encountering the opposite sex stimulus. In males, there was a significant decline in the number of calls after the first minute (Friedman test: *X* ^*2*^ = 8.4, *P* = 0.015), whereas in females the number of USV remained high across the test (Friedman test: *X* ^*2*^ = 3.12, *P* = 0.2) (Fig. 2B).

### Composition

**Fig 3:**
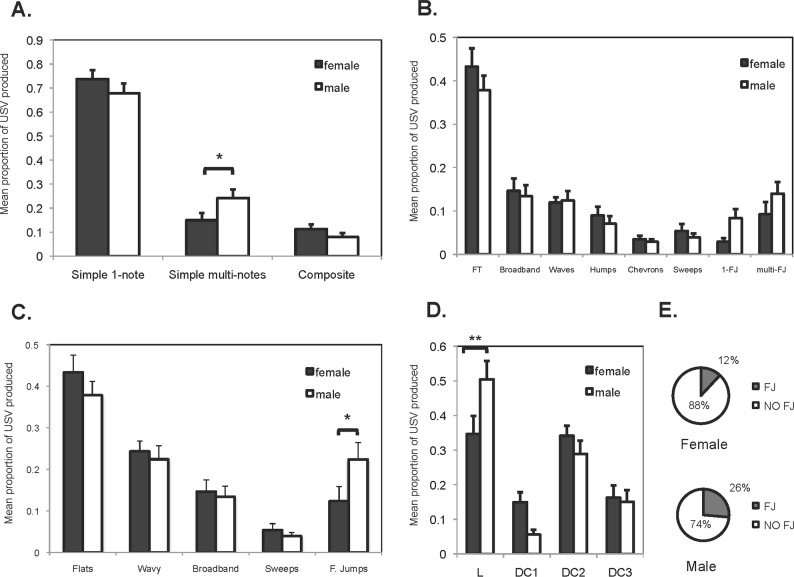
Composition of the vocal repertoire produced by male and estrous female golden hamsters after interacting with an opposite sex individual. **(A)** Proportion of USV produced based on structural complexity (GLM, Complexity*Sex: *F*_*5*,*72*_ = 4.10, *P* = 0.0205). **(B)** Proportion of simple call types produced in each of the 8 major categories (FT = Flats, 1-FJ = one frequency jump, multi-FJ = two or more frequency jumps) (GLM, Call type*Sex: *F*_*15*,*192*_ = 1.54, *P* = 0.156). **(C)** Proportion of simple call types produced merged into 5 major categories. The category Wavy merged the waves, humps and chevrons and the category Frequency Jumps (F. Jumps) merged 1-FJ and multi-FJ types from previous categorization based on 8 categories (GLM, Call type*Sex: *F*_*9*,*120*_ = 2.04, *P* = 0.0925). **(D)** Proportion of USV produced based on different levels of deterministic chaos (L = lineal, DC1 = deterministic chaos 1, DC2 = deterministic chaos 2, DC3 = deterministic chaos 3) (GLM, DC*Sex: *F*_*7*,*96*_ = 4.11, *P* = 0.0087). **(E)** Proportion of USV produced based on the presence or absence of frequency jumps (FJ), (GLM, Sex*FJ: *F*_*3*,*48*_ = 3.62, *P* = 0.0007). *P ≤ 0.05, **P ≤ 0.01.

**Complexity**. Most of the USV produced after a brief opposite sex interaction consisted of 1-note simple calls. These isolated calls were produced 71% of the time. In contrast, calls that showed a higher level of structural complexity, such as those multi-note simple calls and composite calls, were produced less than 24% of the time (Fig. 3A). The proportion of calls observed for each category of complexity was significantly different (GLM: *F*_*5*,*72*_ = 167.8, *P* < 0.01). The majority of USV produced by both males and females were 1-note simple calls, and the interaction between sex and complexity was significant (GLM: *F*_*5*,*72*_ = 4.10, *P* = 0.0205). Males produced a higher proportion of multi-note simple calls (essentially simple calls with frequency jumps) than females (*t*_*1*_ = -2.85 p = 0.0168) (Fig. 3A).

**Call types**. Hamster USV were particularly variable in terms of frequency contours and shapes. We were able to categorize most simple calls into common types already described for other rodents (see Methods). Initially, we found a total of 21 unique call subtypes used in their repertoire within the 3 min recording time (Table 1). The mean individual repertoire size ranged between 9–20 subtypes (mean ± SE = 14.2 ± 0.66). Females showed a significantly larger repertoire size (mean 15.7 ± 0.95) than male subjects (mean ± SE = 12.9 ± 0.79) (*t*_22.5_ = -2.21, *P* = 0.0373). However, female and male repertoires did not differ in diversity according to the comparison of the Shannon-Wiener indices (*t*_23.7_ = 1.13, *P* = 0.269).

A categorization scheme of 21 call subtypes captured most of the variability in frequency modulation observed hamster USV, but it was not practical for identifying general patterns of call type use. Instead of analyzing the repertoire based on many and underrepresented stereotypical call subtypes, we focused on major call types. We performed the statistical analysis at the level of 8 major call types (Table 1). This classification into fewer categories provided a better framework for the general types of frequency contours that can be found in these USV. Close to half of the calls that golden hamsters produced after interacting with an opposite sex individual were flat calls that are characterized mostly by constant frequency. The proportions of vocalizations varied by call type (GLM: *F*_*15*,*192*_ = 40.7, P < 0.001), but the interaction between sex and call type was non-significant (GLM: *F*_*15*,*192*_ = 1.54, *P* = 0.156) (Fig. 3B). However, after being lumped into fewer groups, the probability of occurrence for many of those categories of call types remained quite low (<15%) (Fig. 3B). We then lumped all call types into 5 major groups such that all frequency-modulated calls (waves, humps and chevrons) were merged into one group and all call types that presented frequency jumps were merged into another group (Fig. 3C). Again, close to half of the calls that golden hamsters produced after interacting with an opposite sex individual were flat calls. There was no significant interaction between sex and call type (GLM: *F*_*9*,*120*_ = 2.04, *P* = 0.0925), but the main effect of pairwise comparisons showed a significant difference in the proportion of frequency jump calls produced between males and females (*t*_1_ = -2.82, *P* = 0.0285) (Fig. 3C). Thus, call types with frequency jumps were not produced more or less often than the overall mean response (*t*_4_ = -1.2, *P* = 0.233) but when present, males were more likely to produce them than females. Among the call subtypes with frequency jumps produced (Table 1), the 2-step was the most common subtype emitted by both sexes. Despite the low incidence relative to the whole repertoire, the 1-jump down call subtype represented 20% of the calls with frequency jumps in males and only 8% in females. Thus, 1-jump down may be a call subtype preferentially used by males but rarely produced (<4.45%) after an interaction with an estrous female.

We also observed that part of the sound production system (possibly the vocal tract) performed a high-pass filter (HPF) in some of the USV produced by the hamsters. It was not possible to determine whether there was a filter in 31% of the calls because they were too atonal to show frequency traces. But the lower fundamental frequency was evidently filtered in at least a 24% of the simple calls analyzed. The low frequencies that were filtered ranged between 3 kHz in some calls up to 19 kHz in other calls. However, the most common filter ranged between 12 and 15 kHz, leaving in the spectrogram visible traces of higher harmonics and resonances between 24 and 30 kHz. No significant sex differences were found in the occurrence of HPF.

**Nonlinear phenomena (NLP)**. Hamster USV ranged from tonal (harmonic, periodic, linear) to very noisy (atonal, nonperiodic, nonlinear) sounds. The atonality found in hamster USV could be described as deterministic chaos (DC). DC is characterized by a broadband spectrum with energy at many frequencies and some residual periodic energy caused by nonlinear dynamics in the oscillators that produce the sound [57]. Occasionally, hamster chaotic calls appeared more like a periodic signal with its fundamental frequency and harmonics superimposed on a chaotic signal as if there was a distinction between the sound-generating source and a filter, as commonly observed in mammal vocalizations [58,90,91]. Thus, this chaos observed in hamster USV superficially resembled resonances of the vocal tract known as formants. Usually, the vocal tract enhances and attenuates the transmission of certain frequencies causing the presence of formants [58]. We described the chaotic nature in hamster USV as DC. However, whether the noise observed is produced by nonlinearities of the sound source (e.g. vocal cords) or by resonance of the vocal tract is open to interpretation until the mechanism of production is studied and well understood.

In golden hamsters, nearly half or more of the USV presented some degree of DC (65% of female calls and 50% of male calls were nonlinear) (Fig. 3D). Thus, this type of NLP was a common feature that characterized the USV produced in this species. We found a significant interaction between sex and DC (GLM: *F*_*7*,*96*_ = 4.11, *P* = 0.0087). The main effect pairwise comparison showed a significant difference between sexes only in the proportion of calls categorized as linear (*t*_1_ = -3.19, *P* = 0.0019) and a non-significant tendency in DC1 (*t*_1_ = 2.41, *P* = 0.07) (Fig. 3D). Thus, males are more likely to produce linear and tonal calls than females.

Frequency jumps were another NLP observed in hamster USV that occurred less often than DC. Approximately 12% of female USV and 26% of male USV presented frequency jumps (Fig. 3E). This sex difference was significant (GLM, Sex*FJ: *F*_*3*,*48*_ = 3.62, *P* = 0.0007) suggesting that frequency jumps are found more often in male than in female USV.

Overall, frequency jumps figured in all three analyses of composition. They represented a relatively higher level of complexity, appeared in specific call types and subtypes and can be considered another kind of NLP present in hamster USV. Moreover, these analyses showed that frequency jumps are produced infrequently, but when present, they are preferentially found in male repertoires.

### Acoustic Structure

**Fig 4:**
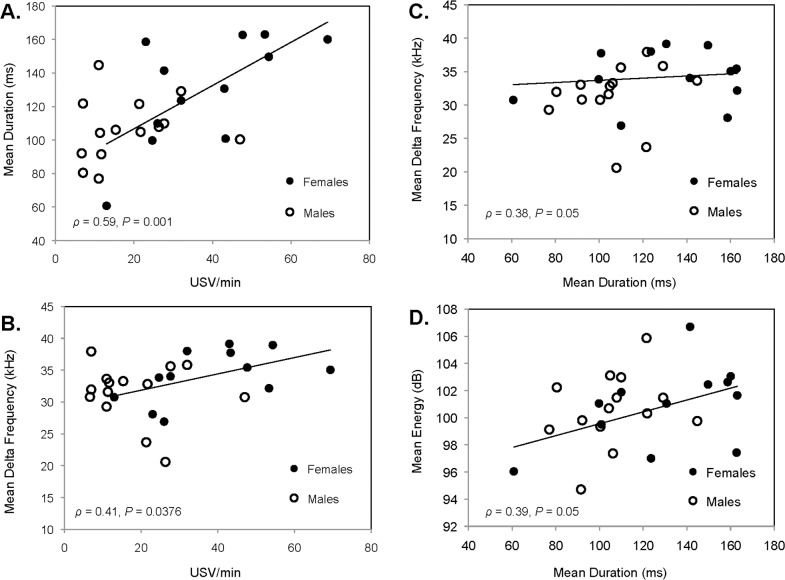
Relationships among acoustic parameters obtained from the analysis of the entire full spectrum of simple 1-note calls produced by both males and estrous females. Spearman correlations between mean calling rates (number of USV/min) and mean duration (ms) **(A)** and frequency bandwidth (kHz) **(B)**. Spearman correlations between mean duration (ms) and mean frequency bandwidth (kHz) **(C)** and mean energy (u) **(D)**.

We analyzed a total of 1103 1-note simple USV from 14 male and 12 female subjects recorded during the post-interaction period following an opposite sex interaction. The PCA retained the first principal components explaining 83.6% of the total variance. However, we did not find significant sex differences between any of the principal components (S1 Table). However, we found that male calls were significantly shorter than female calls (Kruskal-Wallis test: *X* ^*2*^ = 4.89, *P* = 0.027) and had higher minimum frequency (Kruskal-Wallis test: *X* ^*2*^ = 6.61, *P* = 0.0101) (Table 2). As explained in the methods, the acoustic analysis of the full spectrum of the calls included the principal carrier and all its harmonically related and unrelated components (e.g. harmonic frequencies, resonances). However, hamster calls were loud, atonal and characterized by frequent chaos, which often made frequency contours obscure and hard to detect. Thus, it was not surprising to find little information from the parameter extraction. The high variability observed may indicate that these parameters are dynamic and rapidly modulated by the internal state of the caller. In fact, we found that the total number of calls produced by subjects and call duration were predictors of some acoustic parameters. The number of calls per minute was positively correlated with call duration (Spearman *ρ* = 0.59, *P* = 0.001) and delta frequency (Spearman *ρ* = 0.41, *P* = 0.0376) (Fig. 4A,B). When tested separately for each sex, calling rate predicted call duration (males: Spearman *ρ* = 0.29, *P* = 0.3; females Spearman *ρ* = 0.64, *P* = 0.02) and delta frequency (males: Spearman *ρ* = -0.04, *P* = 0.8; females Spearman *ρ* = 0.57, *P* = 0.05) just in females and not in males. Thus, females that called more produced longer vocalizations and of greater bandwidth. Additionally, longer calls showed higher delta frequency (Spearman *ρ* = 0.38, *P* = 0.05) and higher energy (Spearman *ρ* = 0.39, *P* = 0.05) (Fig. 4C,D) in hamsters regardless of sex.

The analysis of the principal carrier was found to be more informative. Only 49% (*N* = 540) of the calls used in the previous acoustic analysis were tonal enough to allow the extraction of the principal carrier and to be analyzed. After a PCA, three principal components were retained explaining 89.7% of the total variance. The first component axis had high loading values for most frequency related parameters, while the third component axis showed high loadings only for energy. The high loading values in the second component were represented by parameters related to IQR bandwidth, entropy and average power (Table 3). We tested the effect of sex on each of the principal components scores obtained and found no significant effect of sex on PC1 or PC3 (Table 3). However, we found a significant effect of sex on the PC2 axis (GLMM: *F*_1,23.6_ = 6.74, *P* = 0.0159), suggesting that the principal carrier of male USV showed lower bandwidth and entropy values. Thus, male calls showed a narrower bandwidth and less disorder of the energy around the frequency carrier. This is also the case if parameters were compared between sexes separately instead of in a multivariate analysis (Table 2).

**Table 3:** Eigen values, percentage of total variation and vector loading values of the acoustic parameters for the first three principal components in the principal component analysis (PCA) for the principal carrier of simple 1-note calls (*N* = 540) (X̅ ± SE).

	PC 1	PC 2	PC 3
Eigen value	6.79	3.24	2.53
% of total variance	48.51	23.15	19.0
Minimum frequency (kHz)	0.659	-0.307	-0.595
Maximum frequency (kHz)	0.933	0.240	-0.115
Q1 Frequency (kHz) ^a^	0.878	-0.151	-0.422
Q3 Frequency (kHz) ^b^	0.938	0.096	-0.314
Center frequency (kHz)	0.920	-0.020	-0.371
Peak frequency (kHz)	0.900	0.007	-0.352
Delta frequency (kHz) ^c^	0.571	0.589	0.406
IQR BW (kHz) ^d^	0.391	0.700	0.221
Average power (dB)	0.547	-0.700	0.384
Peak power (dB)	0.629	-0.522	0.544
Energy (dB)	0.511	-0.384	0.713
F-RMS Amplitude (u) ^e^	0.615	-0.488	0.498
Aggregated Entropy (u)	0.420	0.778	0.307
Average Entropy (u)	0.435	0.700	0.347
Males (n = 14)	0.195 ± 0.369	-0.510 ± 0.273	-0.238 ± 0.201
Females (n = 12)	-0.018 ± 0.254	0.426 ± 0.242	0.293 ± 0.223
*F*-ratio	0.028	6.74	1.76
*P*-value	0.881	0.0159*	0.09

^a^ 1st quartile frequency

^b^ 3rd quartile frequency

^c^ difference between the upper and lower frequency limits of the selection

^d^ inter-quartile range bandwidth

^e^ filtered root-mean-square amplitude.

*F-test significant. Loading values of parameters that loaded strongly to one of the three principal components (values greater than 0.6 are bold typed).

**Fig 5:**
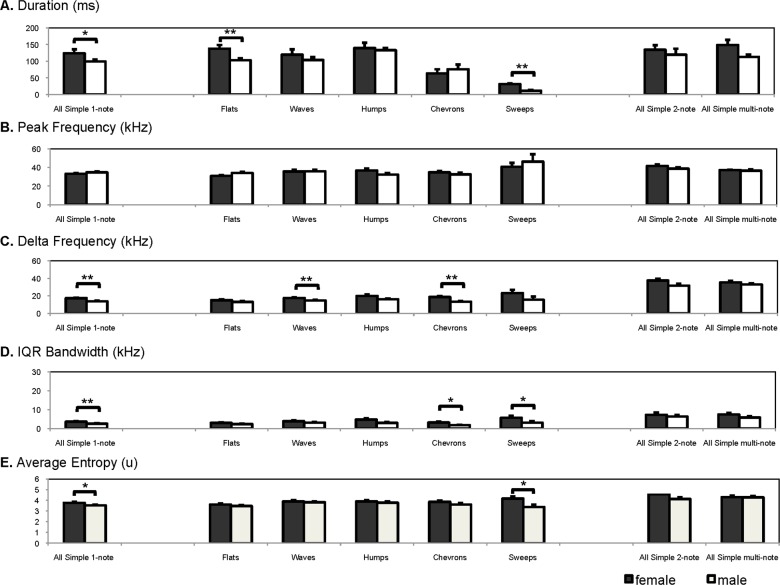
Subset of acoustic parameters for the major categories of call types. Mean acoustic parameters (A-E) of the principal carrier from 5 call types of simple 1-note, all 2-notes combined and all multi-note calls combined in male and estrous female golden hamsters recorded after interacting with an opposite sex individual. *P ≤ 0.05, **P ≤ 0.01.

To examine whether the few sex differences found by the comparison of the PCA were driven by particular call types, such as a distinction between frequency modulated call types like waves, sweeps or humps and constant frequency calls such as flats, we ran separate analyses for each call type and identified and compared the mean values between male and female USV (Fig. 5). Sex differences in duration were found for flat (Kruskal-Wallis test: *X* ^*2*^ = 6.88, *P* = 0.009) and sweep call types only (Kruskal-Wallis test: *X* ^*2*^ = 10.1, *P* = 0.001) (Fig. 5A). There were no significant differences between sexes in peak frequency in any of the call types. Although call types varied in frequency contour, all of them maintained a peak frequency around 30 kHz (Fig. 5B). Frequency bandwidth (delta frequency) was greater for female waves and chevrons (Kruskal-Wallis test: *X* ^*2*^ = 6.26, *P* = 0.01, *X* ^*2*^ = 6.72, *P* = 0.01) while IQR bandwidth was greater for female chevrons and sweeps (Kruskal-Wallis test: *X* ^*2*^ = 4.33, *P* = 0.04, *X* ^*2*^ = 4.52, *P* = 0.03) (Fig. 5C,D). Finally, average entropy was higher only for female sweeps (Kruskal-Wallis test: *X* ^*2*^ = 4.5, *P* = 0.03) (Fig. 5E). Delta frequency, IQR bandwidth and average entropy were not significantly different between sexes in several call types but when call types were pooled together in one analysis these parameters were found to be significantly different between sexes (Table 2, Fig. 5C,D,E). Therefore, sex differences in frequency modulation and entropy were not necessarily attributed to any particular call type.

Simple 2-notes and multi-notes call types were composed of two or more components separated by less than 10 ms of silence. Thus, each note could be analyzed separately. However, we ran acoustic analyses of the whole spectrum including the two or more notes per call to obtain a general idea of the distribution of the main acoustic parameters. We found that 2-notes simple call types (*N* = 149 calls, 11 females, 14 males) were not significantly different in any acoustic parameter between males and females (Fig. 5). Likewise, multi-note call types (*N* = 219, 10 females, 13 males) presented similar acoustic parameters in males and females, and there was no significant tendency for female calls to be longer than male calls (Kruskal-Wallis test: *X* ^*2*^ = 3.46, *P* = 0.06) (Fig. 5A).

**Fig 6:**
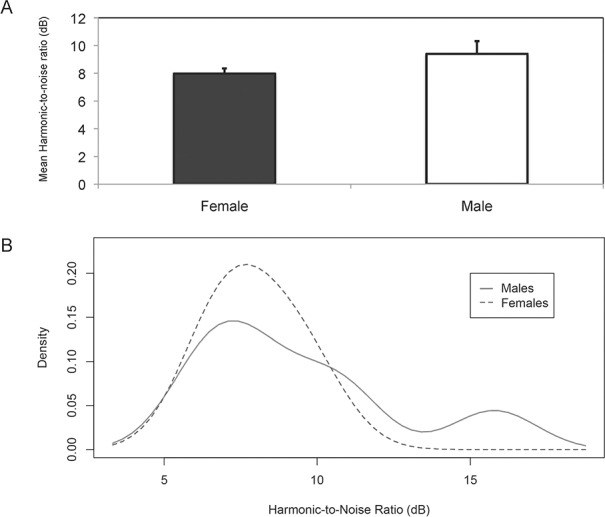
Harmonic-to-noise ratio (HNR) (dB). HNR was quantified from a random subset of 1-note simple calls (*N* = 16) obtained per individual and produced in the post-interaction. **(A)** Mean HNR ± SE in male and in estrous female golden hamsters. **(B)** Kernel density estimation of HNR in males and in estrous females.

**Periodicity (Harmonic-to-noise ratio (HNR))**. The analysis of HNR of randomly selected 1-note simple USV from each individual (n = 16) from the post-interaction period showed no significant differences between sexes (male = 9.4 ± 0.92, female = 7.98 ± 0.37, Log HNR, GLMM: *F*_1,22.7_ = 1.02, *P* = 0.323) (Fig. 6A). The right-skewed distribution (mass distribution concentrated to the left and a long right tail) revealed the prevalence of broadband noisiness (low HNR values) in hamster USV in general (Fig. 6B). However, measures such as skewness (t-test, *t*_22.9_ = 1.07, *P* = 0.296), kurtosis (t-test, *t*_21.1_ = 0.54, *P* = 0.591) or CV (t-test, *t*_22.1_ = -0.64, *P* = 0.537) of male and female HNR distributions did not differ significantly. The HNR values of female USV (without averaging data per individual) ranged between 2.52 and 19.5, while in males they ranged between 2.59 and 29.37. The maximum HNR values were registered in male calls, but only in a few individuals, causing the distribution to have a weak right tail (Fig. 6B). This measure of periodicity agreed with our finding that tonal calls are produced less frequently (Fig. 3D). Both sexes produced atonal and harsh calls more often that were equally aperiodic in terms of HNR.

In addition, in males but not in females, HNR values decreased with call duration (Log HNR, GLMM duration*sex: *F*_1,353.8_ = 8.01, *P* = 0.005). Thus, longer calls were more atonal and noisy in males. Also, in both sexes aggregated entropy (Log HNR, GLMM: *F*_1,347.5_ = 368, *P* < 0.001), average entropy (Log HNR, GLMM: *F*_1,346.3_ = 514.8, *P* < 0.001) and IQR bandwidth (Log HNR, GLMM: *F*_1,355.9_ = 161.5, *P* < 0.001), were negatively correlated to HNR, demonstrating that higher aperiodicity (lower HNR values) can be predicted by increased entropy and bandwidth. Calling rates and HNR values were not significantly correlated (Log HNR, GLMM: *F*_1,20.9_ = 0.43, *P* = 0.5).

**Individual variability**. We found that all acoustic parameters varied more within than among individuals. Their PIVs were less than 1 and none of them could serve as a cue for individual identity when evaluated separately (Table 4). In order to have all parameters considered in one model, we used the stepwise forward DFA to identify those call parameters that could contribute the most to the discrimination of individuals. The model retained 6 of the 14 parameters. Average power, average entropy, delta frequency, Q1, energy and aggregated entropy were found to be parameters that contributed the most to discriminating among individual callers. However, the DFA yielded an average of 43.8% of correct assignment to individuals of the original data and the cross-validation showed a lower percentage of correctly classified calls (38.2%). Therefore based on the acoustic parameters measured, USV were not sufficiently distinct among individuals. HNR was not included in the DFA given that this measure was obtained for only a subset of vocalizations. However, HNR was not an individually distinct characteristic (PIV<1) and, within the vocal repertoire of any given individual, it was common to observe USV of different tonality.

**Table 4:** Coefficient of variations (X̅ ± SD) of call parameters of the principal carrier of simple 1-note calls (N = 523) produced by male (*N* = 11) and female (*N* = 12) subjects after interacting with a stimulus hamster of the opposite sex.

	Mean CV_w_	Mean CV_a_	Mean PIC (CV_a_/CV_w_)
Duration (ms)	48.8 ± 15.3	30.2	0.68
Minimum frequency (kHz)	21.3 ± 7.99	13.1	0.69
Maximum frequency (kHz)	20.3 ± 6.42	8.51	0.46
Q1 Frequency (kHz) ^a^	18.1 ± 7.40	10.4	0.66
Q3 Frequency (kHz) ^b^	18.8 ± 6.96	9.21	0.55
Center frequency (kHz)	18.6 ± 7.28	9.64	0.59
Peak frequency (kHz)	19.1 ± 7.36	9.59	0.57
Delta frequency (kHz) ^c^	38.2 ± 10.7	18.5	0.52
IQR BW (kHz) ^d^	66.9 ± 16.9	31.9	0.50
Average power (dB)	7.34 ± 1.62	5.14	0.74
Peak power (dB)	6.74 ± 1.48	4.49	0.71
Energy (dB)	6.21 ± 1.40	3.84	0.66
F-RMS Amplitude (u) ^e^	67.1 ± 17.3	42.6	0.70
Aggregated Entropy (u)	15.9 ± 4.33	7.99	0.53
Average Entropy (u)	14.6 ± 4.53	8.55	0.65
Harmonic-to-Noise Ratio (HNR) (dB)	38.0 ± 11.6	29.8	0.85

^a^ 1st quartile frequency

^b^ 3rd quartile frequency

^c^ difference between the upper and lower frequency limits of the selection

^d^ inter-quartile range bandwidth

^e^ filtered root-mean-square amplitude. CV_w_ = coefficient of variation within-individuals, CV_a_ = coefficient of variation among-individuals, PIC = Potential for individual coding.

## Discussion

Golden hamsters produced a large and variable number of USV during social encounters. Opposite sex encounters elicited the greatest number of vocalizations after the stimulus animal was removed, suggesting that these calls may be described as socially elicited calls produced in a sexual context. Calling rates by most males and females were high but there was substantial individual variability. The vocal repertoire was mainly composed of structurally 1-note simple calls, classified as flats, and at least half of them presented nonlinear aperiodicity that could be classified as some degree of deterministic chaos. The prevalence of this aperiodicity was confirmed by low values of HNR for most calls. Composite calls and multi-note calls (call types involving frequency jumps) occurred less frequently overall. Males were more likely to produce tonal calls and call types with frequency jumps than females. Overall, male calls were found to be shorter than female calls. High calling rates predicted longer call duration and higher delta frequency in both sexes. Call duration, in turn, was positively correlated with energy and delta frequency in both sexes and negatively correlated with HNR but only in males. The analysis of the principal carrier of 1-note simple calls revealed that most of the variation (71.7%) fell between two major PC factors, one comprising frequency and amplitude parameters and another representing scores of entropy and bandwidth. Sexes were significantly different in the second axis with male calls showing lower entropy and bandwidth values. Finally, the variation within individuals was not higher than the variation among individuals obtained for call features analyzed independently. A discriminant function analysis revealed that, indeed, calls were hard to discriminate statistically among individuals.

### Vocal repertoire in golden hamsters: commonalities and differences with other rodents

Based on our criteria for classification of structural complexity, hamster USV seemed to lack high complexity. Most calls appeared to be produced in isolation and not in obvious or specific sequences or bouts. We observed calls that were close enough in time to be categorized not as independent calls but as a composite call. But these were produced rarely and no particular temporal associations were obvious. To determine whether golden hamster USV exhibit predictable sequences or bouts requires further call type temporal analysis as has been done in the house mouse [26,33].

We observed a relatively higher level of complexity when frequency jumps were present. However, call types with frequency jumps (multi- note call types) were produced in a significantly lower proportion than other call types. A high occurrence of simple calls and a low occurrence of USV with frequency jumps have also been reported in rats and mice. For instance, 48% of the USV that a male rat produced after the separation from two cage mates, were flat calls and less than 13% were calls with frequency jumps [68]. Similarly, male mice emitted less than 20% of calls with frequency jumps when exposed to a female [92] or just 5% when exposed to female urine [93]. Hanson & Hurley [31] found a very low percentage of frequency jumps produced by male mice during mating and after female removal. Interestingly, Hammerschmidt et al. [46] recorded a lower proportion of frequency jumps in USV produced in response to anesthetized compared to awake intruders. Also, frequency jumps have been found to be more common in pup isolation calls than in adult social calls [33]. Thus, sudden jumps in frequency seem to be part of the vocal repertoire of most species of rodents, including hamsters, and the low probability of occurrence may be non-random and deserving of further study.

USV produced by adult hamsters following intersexual encounters were characterized by remarkable variability. High acoustic variability has been noted in all other studies that have examined and categorized ultrasonic vocal repertoire in rodents, both in mice pups [80] and in adult rodents (e.g. [31,37,68,92,93]). We classified hamster USV into call types, but these were far from stereotypical vocalizations. Instead, call types or “syllable” types used in other rodents served as a reference point to categorize hamster USV into major groups with specific characteristics. In hamsters, the frequency trajectory determined the category for each call based on the assumption that different frequency trajectories would be perceptually different to a hamster. Call types characterized by increasing and/or decreasing frequency (waves, humps, chevrons and sweeps) were produced less frequently than constant frequency, flat calls (Fig. 3B). A higher occurrence of constant frequency calls has also been reported in rats. A brief separation from a cage mate elicited high rates of flat 50-kHz calls in rats that stayed in the home cage [68,94]. Amphetamine administration decreased the proportion of flat calls and increased a rat-specific type of frequency-modulated (FM) call (the trill) [68]. Burgdorf et al. [53] found that rats, before mating and during rough-and-tumble play behavior, produced FM 50-kHz calls (trills and multi-step calls), more that flat 50 kHz calls. In fact, rats self-administered playback of FM 50-kHz calls and avoided flat 50 kHz calls. Thus, it has been proposed that rats use flat calls to maintain or reestablish social contact in a non-rewarding or neutral context, and that FM calls are associated with appetitive stimuli involved in initiation and maintenance of positive social behaviors [53,68]. Although we cannot determine whether flats calls in hamsters are related to a specific affective situation other than the separation from an opposite sex hamster, flats calls may represent the “default” type to produce when vocal production is elicited. The occurrence of calls of varying frequency modulation or sudden frequency jumps may increase during initial sexual contact or, in the case of males, in the presence of a lordotic female.

Although, we observed some similarities in composition between golden hamsters and male mice when exposed to female odors [93], or females [31,92] or male rats when separated from a cage mate [68], several features distinguished hamster USV from the rest of rodent USV. Frequency ranges in hamsters were typically between 20–55 kHz and peak frequencies between 33–37 kHz, thus not as high in frequency as in most USV observed in the house mouse or in the rat. Hamsters did not show evidence of trills, as described in rats, or a high proportion of sweeps, as reported for mice [31,92]. In contrast to other rodents, hamsters produced a greater proportion of calls that were broadband, noisy and of chaotic energy distribution in which the extraction of the principal carrier was not possible (Fig. 3D). Calls with defined frequency contour were often accompanied by noise and atonality. This particular feature of hamster USV has not been noted to be common in other adult rodent calls. Grimsley et al. [33] recorded pup isolation and adult social vocalizations in mice, and compared the number and proportion of different syllable types across age. Most syllables were tonal but some presented subharmonics and deterministic chaos. They reported that nonlinearities in USV became progressively more common from p5 to p13 (0.3–23.5%) but were rare in adult syllables (only 1.6%). In golden hamsters, pup isolation calls have been described as broadband with harmonic independent components or as narrow-band with harmonic dependent components [95] and of wider frequency bandwidth compared to other hamster species [96]. Thus, nonlinearities are likely present in pup golden hamster USV and it would be interesting to examine the development of these features into adulthood.

### Sex and individual differences in golden hamster USV

In this study we focused on the comparison of the vocalizations produced by males and females within the same sexual context and found no evidence for sex-specific vocalizations. Instead, we found some sex-typical characteristics in the vocal repertoire. Males were more likely to produce vocalizations with frequency jumps than females and females showed a larger, but not more diverse, repertoire size than males. The presence of noise and chaos was a prominent characteristic that appeared in half of the calls produced by both sexes, but the probability of occurrence depended on sex. Male produced more calls that were tonal and less chaotic. Again, the incidence of frequency jumps, which are also considered NLP, was found to be uncommon but when present, was more likely to be found in male calls. These acoustic characteristics have occasionally been found in the vocalizations of males and females of other species and whether they can be used for sex discrimination and recognition remains to be tested [34,67]. Instead, many studies have raised hypotheses to explain the potential significance of the NLP in vocal communication (see below).

Floody et al. [7] had previously found that golden hamsters vocalized to a great extent during and after heterosexual interactions, and found a sex difference in calling rates [7,20]. In the present study, we also found that females were highly motivated to vocalize after a male was removed and maintained high rates during most of the recording period, whereas males’ calling rates rarely reached high rates and declined faster. Average post-interaction calling rates were not quite significantly different between sexes. Male calling rates may have been lower than female calling rates because during the interaction period across the mesh, the female went into the lordosis position and remained in this position for most of the 3 min recording time. During this period, most males vocalized extensively (data not shown). Thus, by the time the post-interaction period was recorded, males had already been calling during the previous 3 min and may have experienced some fatigue. In contrast, females, which do not vocalize while in lordosis [7,21] and presumably had not vocalized much during the interaction, may have directed more energy into calling during the post-interaction period. This could explain why female USV were longer than male USV. Undoubtedly most individuals of both sexes exhibited high motivation to vocalize during this context.

The acoustic structure of the golden hamster’s USV produced by both sexes following an opposite sex interaction was previously found to be similar in dominant frequency and total duration [76]. However, male calls presented fewer rapid changes in amplitude and frequency than female calls [76]. After a more thorough acoustic analysis and a larger sample size, we found a no significant sex difference in PC1, an axis mostly represented by frequency parameters, which agrees with previous findings. PC2 represented by entropy, average power and frequency bandwidth was significantly different between sexes, but represented only a small proportion of the whole multivariable variation that described simple vocalizations (23% of the total variation). Moreover, we did not find sex differences in the HNR of the calls. Most male and female USV exhibited low values of HNR (high aperiodicity), but males represented mostly the high end of the HNR scale.

Overall, it is difficult to know whether the modest sex differences found in composition and acoustic structure could function in sex discrimination and recognition. However, we cannot rule out that functional possibility. Male giant panda vocalizations (bleats) had higher frequency modulation and jitter than females when acoustic features were analyzed separately [97]. Female mice produced USV that were longer, with wider frequency bandwidth and more jumps than male mice during an extended opposite sex interaction across a contact window [34]. These results are somewhat similar to the sex differences we found in this study with the exception of the higher occurrence of frequency jumps in female calls. However, no sex differences in rodent USV have been found in two other studies. The acoustic structure and call type usage of USV produced by a male or a female resident in response to a female intruder (of uncontrolled estrous state) were not significantly different in house mice [46]. California mice produce USV when alone or in the presence of another conspecific in the wild, but the vocalizations were found to lack sexual dimorphism [45]. In any case, even when statistically significant sex differences are found, male and female distributions often overlap, and biological significance cannot be assumed without an empirical test [98].

Although mice can discriminate different types of USV [99], to our knowledge no study has examined whether rodents can perceptually differentiate among individuals using USV. Socially elicited USV have been reported to be individually distinct in mice [26,31,38]. The variation that we found in the acoustic parameters rejected the hypothesis that hamster USV could be identified statistically as individually distinct. Although this question should be answered with more recording sessions per individual as opposed to one, such as in this study, this first analysis does not suggest that individual signatures will be found. It is possible that a different set of acoustic parameters may lead to different results. Thus there is no evidence yet that USV in hamsters could be used in individual recognition. Golden hamsters are well known for individual recognition of familiar conspecifics and kin using odors from various body sources [100,101]. It is possible that this is the main channel of communication when hamsters first meet and become familiar with each other. So far, we have not recorded spontaneous calling in the colony and, in general, USV have short-range capabilities and are not designed to travel far [102,103]. Instead, USV in hamsters are elicited in response to social signals such as odors, playbacks of USV and when a conspecific is out of sight after an encounter, as shown in the present study [7,104]. Thus, USV in hamsters may be conveying other information than individual identity.

### Potential relevance of noise, chaos and high variability in vocal communication

The evident complexity in the hamster USV repertoire is most likely the result of variations produced by the vocal production mechanism. Although, the mechanism of ultrasound production in rodents it is still not well understood [103,105], evidence has demonstrated that laryngeal muscles are necessary for ultrasound production [106–109]. It was recently found that rat USV call types are the result of the coordination of laryngeal and vocal tract muscles in combination with the respiratory system [109]. It is unknown, however, whether all the variability found in frequency modulation and the existence of nonlinear phenomena (NLP) in hamster USV can be explained by laryngeal muscle activity and subglottal pressure. At the very least, deterministic chaos is considered to be the result of the desynchronization of oscillators that produce the sound (e.g., vocal folds) caused by increased vocal effort [57,82]. Frequency jumps are another instability of the vibratory regime observed when the vibration rate moves up and down abruptly, discontinuously causing sudden changes in the fundamental frequency [81]. Interestingly, as sublaryngeal pressure increased in the larynx of an ultrasonic frog, bifurcations began to occur that led eventually to chaos [64]. Therefore, there may be differences in the amount of effort required to produce different call types, frequency jumps and in the incorporation of NLP based on the coordination of respiration, larynx and vocal tract. Thus, chaos and frequency jumps in hamsters may indicate elevated vocal effort.

The finding that NLP is ubiquitous in animal vocalizations has increased the interest in understanding these phenomena [57]. Although nonlinearities in the vocal repertoire of a species may be just a byproduct of the vocal system, rather than an evolved signaling trait, the number of studies finding a functional role for these phenomena in listener perception has increased in recent years, with three types of evidence suggesting functional hypotheses [57]. First, NLP have been found to convey information about the arousal state of the caller. Most of the vocalizations produced during high affect contexts and distress in infant elephants exhibited nonlinear chaos, lower tonality (lower HNR values) and longer duration than those vocalizations produced in situations of presumed lower levels of arousal [61]. Low tonality in calls is also found in adult subordinate elephants in the presence of dominants [110] and in young rhesus macaques during distressful situations [111]. Second, sudden introduction of chaos and abrupt changes in frequency may be considered as features designed to generate unpredictability that function to prevent habituation to the caller and increase responsiveness [57]. For instance, meerkats spent less time foraging in response to playbacks of natural nonlinear than linear alarm calls [62], and nonlinear alarm calls required longer to habituate [112]. Also, male harsh roars (nonlinear), and not common roars (linear), increased females’ attention to playbacks and the level of response to subsequent roars in red deer [113]. Third, irregular chaotic vibrations produce a loud broadband spectrum of frequencies that enhances the projection of formants and provides information about the unique filtering pattern associated with the vocal tract [57]. An individual’s vocal tract is closely correlated with body size and quality in some species [57,114]. These hypotheses of the functional significance of NLP are not mutually exclusive and could provide insight about the utility that chaos and frequency jumps may have in hamster USV. In mammals, most studies examining the role of NLP or simply vocal complexity have centered on antipredator vocal strategies, mother-offspring communication and male-male competition [59] and less on inter-sexual selection [113].

Based on the patterns of within-individual variability found, we believe that most acoustic features in hamster USV are dynamic signals. Dynamic signals change greatly from call to call in a matter of seconds depending on the social context and are characterized by high CV_w,_ whereas static signals are more stable, showing low CV_w_, and are more likely to provide information about the long-term condition of the caller [115,116]. Typically, call rate, duration and amplitude are dynamic cues that can change rapidly depending on the urgency of the situation and reflect the current state of the caller [113,115,117,118]. Mice that were housed in isolation showed higher social motivation by emitting more and longer USV and spending more time in contact during a social interaction than mice that were housed in groups [70]. Perhaps hamsters that were more sexually motivated produced more USV that were also longer and of greater bandwidth (Fig. 4). As discussed above, males may have produced calls that were more tonal, shorter, of narrower bandwidth and lower entropy than females as a result of male fatigue, except for the higher incidence of calls with frequency jumps that may have required higher vocal effort. Although static and dynamic signals are just two ends of a continuum, dynamic signals may be giving honest information about an individual’s current motivational state, physiological condition, and stamina, as has been found in other mammal species (e.g. [114,118,119,120], while static signals may serve as more sender identity parameters [118]. Further research needs to be done to examine the role that these acoustic parameters may play in different contexts varying in the level of situational urgency or sexual motivation.

## Conclusions

The high calling rates, the high incidence of chaos, the low periodicity (low HNR values) and the high within-individual variability found in this study suggest that these vocalizations are dynamic signals that are probably modulated by the context (external factors) and the internal state of the caller (internal factors). Although the modest sex differences that we found may be the result of sexual selection, they may also be the product of a sex difference in motivation and current state of the caller. Hamster USV may function as an index of motivational state, current condition (stamina) and level of sexual arousal of the caller. The unpredictability of its natural nonlinear complexity may facilitate and maintain the receiver’s attention. Regardless of the information that hamster USV may be transmitting, it will be important to understand how this commonly produced signal during social interactions can influence the behavioral and physiological responses of the receiver.

## Supporting Information

S1 Table Eigen values, percentage of total variation and vector loading values of the acoustic parameters for the first three principal components in the PCA for the full spectrum of simple 1-note calls (X̅ ± SE). (DOCX)
