# Supplementary material for: Ultrasonic Vocalizations in Golden Hamsters (Mesocricetus auratus) Reveal Modest Sex Differences and Nonlinear Signals of Sexual Motivation
Source: PLoS One. 2015 Feb 25;10(2):e0116789. doi: 10.1371/journal.pone.0116789 (PMC4340904; doi:10.1371/journal.pone.0116789)
Supplement: S1 Table — (DOCX) [file pone.0116789.s001.docx]

**Table S1.** Eigen values, percentage of total variation and vector loading values of the acoustic parameters for the first three principal components in the PCA for the full spectrum of simple 1-note calls (± SE).

|  | PC 1 | PC 2 | PC 3 |
| --- | --- | --- | --- |
| Eigen value | 5.07 | 3.82 | 2.8 |
| % of total variance | 36.2 | 27.3 | 20.0 |
| Minimum frequency (kHz) | -0.090 | 0.447 | **0.676** |
| Maximum frequency (kHz) | **0.805** | -0.329 | -0.095 |
| Q1 Frequency (kHz) ^a^ | **0.629** | 0.380 | 0.594 |
| Q3 Frequency (kHz) ^b^ | **0.816** | -0.141 | 0.475 |
| Center frequency (kHz) | **0.770** | 0.148 | 0.571 |
| Peak frequency (kHz) | **0.692** | 0.194 | 0.547 |
| Delta frequency (kHz) ^c^ | **0.688** | -0.484 | -0.500 |
| IQR BW (kHz) ^d^ | 0.366 | **-0.656** | -0.064 |
| Average power (dB) | 0.418 | **0.802** | -0.314 |
| Peak power (dB) | 0.517 | **0.669** | -0.475 |
| Energy (dB) | 0.566 | 0.447 | **-0.636** |
| F-RMS Amplitude (u) ^e^ | 0.556 | **0.607** | -0.439 |
| Aggregated Entropy (u) | 0.546 | **-0.714** | -0.115 |
| Average Entropy (u) | 0.552 | **-0.680** | -0.170 |
| Males (n=14) | 0.005 ± 0.26 | 0.266 ± 0.347 | -0.238 ± 0.201 |
| Females (n=12) | -0.094 ± 0.26 | -0.181 ± 0.241 | 0.293 ± 0.223 |
| *X^2^* | 0.02 | 0.518 | 2.88 |
| *P-*value | 0.874 | 0.471 | 0.0896 |

^a^ 1st quartile frequency, ^b^ 3rd quartile frequency, ^c^ difference between the upper and lower frequency limits of the selection, ^d^ inter-quartile range bandwidth, ^e^ filtered root-mean-square amplitude. Loading values of parameters that loaded strongly to one of the three principal components (values greater than 0.6) are bold typed.
